# Exploring the Influence of Daily Climate Variables on Malaria Transmission and Abundance of *Anopheles arabiensis* over Nkomazi Local Municipality, Mpumalanga Province, South Africa

**DOI:** 10.1155/2018/3143950

**Published:** 2018-10-09

**Authors:** Gbenga J. Abiodun, Kevin Y. Njabo, Peter J. Witbooi, Abiodun M. Adeola, Trevon L. Fuller, Kazeem O. Okosun, Olusola S. Makinde, Joel O. Botai

**Affiliations:** ^1^Research Unit, Foundation for Professional Development, Pretoria, South Africa; ^2^Department of Mathematics and Applied Mathematics, University of the Western Cape, Private Bag X17, Bellville 7535, South Africa; ^3^Institute of the Environment and Sustainability, University of California Los Angeles, Los Angeles, California, USA; ^4^South African Weather Service, Private Bag X097, Pretoria 0001, South Africa; ^5^School of Health Systems and Public Health, Faculty of Health Sciences, University of Pretoria, Pretoria, South Africa; ^6^Department of Mathematics, Vaal University of Technology, X021, Vanderbijlpark 1900, South Africa; ^7^Department of Statistics, Federal University of Technology, P.M.B 704, Akure, Nigeria; ^8^Department of Geography, Geoinformation and Meteorology, University of Pretoria, Private Bag X20, Hatfield 0028, South Africa

## Abstract

The recent resurgence of malaria incidence across epidemic regions in South Africa has been linked to climatic and environmental factors. An in-depth investigation of the impact of climate variability and mosquito abundance on malaria parasite incidence may therefore offer useful insight towards the control of this life-threatening disease. In this study, we investigate the influence of climatic factors on malaria transmission over Nkomazi Municipality. The variability and interconnectedness between the variables were analyzed using wavelet coherence analysis. Time-series analyses revealed that malaria cases significantly declined after the outbreak in early 2000, but with a slight increase from 2015. Furthermore, the wavelet coherence and time-lagged correlation analyses identified rainfall and abundance of *Anopheles arabiensis* as the major variables responsible for malaria transmission over the study region. The analysis further highlights a high malaria intensity with the variables from 1998–2002, 2004–2006, and 2010–2013 and a noticeable periodicity value of 256–512 days. Also, malaria transmission shows a time lag between one month and three months with respect to mosquito abundance and the different climatic variables. The findings from this study offer a better understanding of the importance of climatic factors on the transmission of malaria. The study further highlights the significant roles of *An. arabiensis* on malaria occurrence over Nkomazi. Implementing the mosquito model to predict mosquito abundance could provide more insight into malaria elimination or control in Africa.

## 1. Introduction

Malaria is a devastating vector-borne disease dominant in most tropical countries especially sub-Saharan Africa. In South Africa, it is found in the northeastern part of Mpumalanga Province, KwaZulu-Natal Province, and Limpopo Province. There has been a noticeable increase in malaria over the three provinces lately. For instance, during March 2017, a total of 9478 malaria cases and 76 deaths were reported over South Africa compared to 6385 malaria cases and 58 deaths in the 2015/16 season [1]. Of this, a total of 1,790 cases were confirmed in Mpumalanga Province between April and June 2017 [1]. The upsurge has been linked to climatic and environmental factors and a reduction in indoor residual spraying (IRS) in areas where malaria cases had declined in recent seasons [1]. Malaria in Mpumalanga Province is seasonal, beginning with the first rainfalls in October, getting to a peak in January, and remaining high until May. Nevertheless, transmission is still unstable and liable to intermittent outbreaks [2]. *Plasmodium falciparum* is the major parasite and transmitted mainly by *Anopheles arabiensis* and *Anopheles funestus* vectors [3, 4]. Malaria distribution is mainly in the low-lying areas (Nkomazi, Bushbuckridge, Mbombela, Umjindi, and Thaba Chweu local municipalities) bordering Swaziland and Mozambique, with suitable climate conditions for malaria transmission [2, 5]. Of all the municipalities, Nkomazi has been mapped out as the most epidemic region in the province. The *An. arabiensis* occurs in all the three malaria-endemic provinces of South Africa; however, it is known to be the dominant vector in Nkomazi Municipality [4]. The *An. arabiensis* mainly breeds in small, sunlit, temporary, fresh-water pools, but occasionally, breeding sites are also found along the margins of dams in cattle hoof prints as well as in rice paddies [6]. They can be found resting indoors and outdoors with adult females feeding on both humans and animals, particularly cattle [6]. On the contrary, the *An. funestus* breeds in permanent and semipermanent fresh-water pools or slow-moving streams containing emergent vegetation, such as swamps, large ponds, and lake edges [6]. As reported by Adeola et al. [7] within the same study area as this current study, the vectors are found to be high in areas with the normalized difference vegetation index (NDVI) between 0.41 and 0.50. These are dominantly areas under intensive irrigation which cover about 18% of the total land area [5].

Although several studies have investigated the impact of climate on malaria prevalence in South Africa, only a few have focused on Nkomazi—one of the more significant epidemic regions in South Africa [5]. More importantly, the importance of mosquito abundance on malaria transmission is often overlooked. For instance, the malaria cases over a 30-year period were analyzed over KwaZulu-Natal [8]. The findings identified daily maximum temperatures and rainfall as the primary climatic factors responsible for malaria transmission over the province. The impact of climate change was further investigated through climate-based mathematical models [9]. Similarly, other studies [10, 11] have highlighted the importance of temperature and rainfall on malaria over the same province. Results in the paper by Komen et al. [12] revealed a strong correlation between temperature and malaria prevalence in Limpopo. Furthermore, Ikeda et al. [13] investigated the relationship between malaria incidence and spatiotemporal climate and found significant associations between incidence anomalies and climate patterns. Most of these studies are not fine-scaled analysis but general across the regions. An in-depth analysis is warranted to better understand transmission factors and control [14].

The relationship between malaria cases and three possible drivers (rainfall, geography, and source of infection) was explored over Mpumalanga Province [2]. The latter study highlighted the significance of rainfall on malaria and further concluded that malaria incidence follows rainfall with a lag of one month. Using the seasonal autoregressive integrated moving average (SARIMA) model, Adeola et al. [5] considered climatic and environmental factors (rainfall, temperature, humidity, vegetation indices, and water index) to investigate malaria cases over Nkomazi Municipality. This implies that factors influencing malaria vary by provinces across South Africa [5]. The outcome of the study highlighted the importance of these factors and indicated the time lag requirement for transmission [2]. However, the interconnection between these factors and malaria was not deeply considered in these studies. Furthermore, *Anopheles arabiensis* has been identified as the major malaria vector over the study region; however, the dynamics of the vector was overlooked in the study of Adeola et al. [5]. The present study intends to use new methods to cover some of these gaps. For example, wavelet analysis will be used to examine the interconnectedness between the climate variable, mosquito abundance, and malaria occurrence. A new climate-based mathematical model will be used to simulate the mosquito population dynamics of the study region. In particular, this study aims to do a fine-scale in-depth investigation of the impact of climate daily variables on mosquito abundance and malaria occurrence over Nkomazi Local Municipality and hence extrapolation to other regions. The interconnections between these variables as well as the time lag between the variables and malaria transmission will also be established.

## 2. Materials and Methods

### 2.1. Study Area

The Nkomazi Local Municipality is located in the eastern part of Ehlanzeni District of Mpumalanga Province. The municipality is strategically positioned in a corner between Swaziland (north of Swaziland) and Mozambique (west of Mozambique). It is the smallest of the four municipalities in the district, making up 17% of its geographical area. Nkomazi climate is subtropical with an average annual temperature of 28°C and the rainfall average of 775 mm [5]. The driest month is July, with 9 mm of rainfall. The highest amount of rainfall is received in January, with an average of 127 mm. Also, the warmest month of the year in Nkomazi is January with an average temperature of 26.2°C, while the lowest average temperatures in the year occur in June, when it is around 18.4°C. The municipality is known for sugarcane production, and irrigation is commonly used. It is a high-risk malaria region with an incidence rate of about 500 cases per 100,000 [2, 5], which makes the municipality the most significant endemic area in Mpumalanga Province.

### 2.2. Data Compilation and Analysis

The daily rainfall (mm) derived from the Tropical Rainfall Measuring Mission (TRMM) using the Mirador platform of National Aeronautics and Space Administration (NASA) was used in this study. The rainfall estimate is a gridded product with a spatial resolution of 0.25° × 0.25°. Similarly, the temperature and relative humidity data were extracted from the National Centers for Environmental Prediction (NCEP) Climate Forecast System Reanalysis (CFSR). The dataset which is now available from January 1979 to March 2017 was initialized 4 times per day (0000, 0600, 1200, and 1800 UTC), and the 6-hourly atmospheric, oceanic, and land surface analyzed products are available at 0.5, 1.0, 1.9, and 2.5° horizontal resolutions [15]. The 6-hourly climate dataset was converted to daily with 0.5° × 0.5° resolution for the purpose of this study.

It is essential to consider a long-term data series when assessing the impact of climate variability on malaria transmission over a region [14]. This is equally applicable when investigating the variability of mosquito populations. However, this becomes challenging in the absence of long-term mosquito data. To overcome this challenge, several studies [16–20] have used the deterministic mosquito model to simulate mosquito abundance over some regions. Likewise in this study, due to the unavailability of mosquito data over Nkomazi, the dynamic mosquito model presented by Abiodun et al. [17] was used to simulate the abundance of *An. arabiensis* over the study region. To examine the robustness of the model, the climate-based mosquito model (mainly designed for *An. arabiensis—*the major vector across South Africa) consisting of both aquatic stage and adult stage mosquitoes was validated over a town in eastern Sudan. In the present study, we use the climate data of the five villages representing Nkomazi Local Municipality as the driving tools of the model. For further details on the model, we refer readers to the study by Abiodun et al. [17].

**Figure 1:**
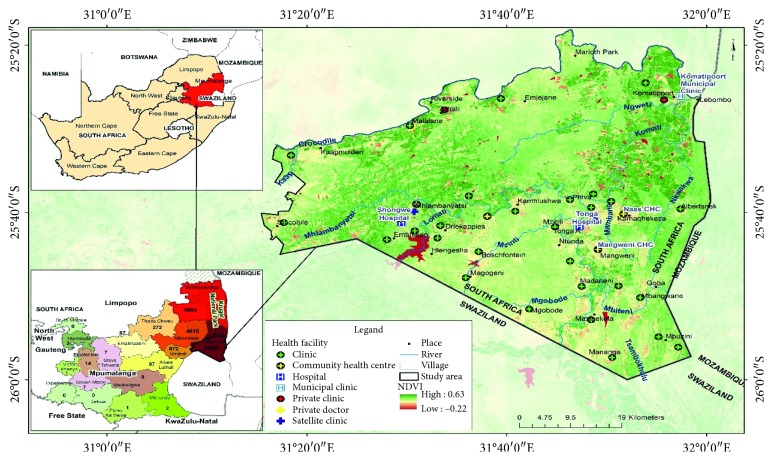
Location of the study area, showing the villages and health facilities (after Adeola et al. [5]).

Daily malaria data from January 1997 to December 2015 were considered for this study. The data were sourced from the provincial Integrated Malaria Information System (IMIS) of the Malaria Control Programme in the Mpumalanga Provincial Department of Health with ethical approval number (MP_2014RP39_978). The locally recorded cases with minimal imported cases were extracted from clinics and hospitals of five epidemic villages in Nkomazi Local Municipality. The villages and their corresponding health facilities include Komatipoort (Komatipoort Hospital), Tonga (Tonga Hospital), Mangweni (Mangweni CHC), Matsamo (Shongwe Hospital), and Kamaqhekeza (Naas CHC), as shown in Figure 1.

### 2.3. Wavelet Coherence Analysis

We used wavelet coherence analysis to investigate the interconnectedness between climate variables, mosquito abundance, and malaria transmission over Nkomazi Local Municipality. The choice of this approach is based on its ability to identify simultaneously the time intervals and the frequency bands, where two time series are correlated. Although the technique is based on the logic of Fourier analysis, it addresses the later limitations by using different scales to analyze different frequencies, contrary to the Fourier analysis that uses the same scale for all frequencies. As a result, the wavelet transformation can use good frequency resolution and poor time resolution at low frequencies, as well as good time resolution and poor frequency resolution at high frequencies. Furthermore, the approach has been identified as a most efficient method among the various methods developed to study nonstationary data [21–23]. The methodology has also been considered in many fields such as atmospheric sciences, geophysics, and climatology [24, 25], and epidemiology [26–28]. As defined in Fourier analysis, the univariate wavelet power spectrum can be broadened to analyze statistical relationships between two time series *x*(*t*) and *y*(*t*) by computing the wavelet coherence, using the following formula:

(1) $$\begin{array}{c} R_{x,y}\left(f,\tau \right)=\frac{\left|\langle W_{x,y}\left(f,\tau \right)\rangle \right|}{{\left|\langle W_{x}\left(f,\tau \right)\rangle \right|}^{1/2}\cdot {\left|\langle W_{y}\left(f,\tau \right)\rangle \right|}^{1/2}}, \end{array}$$

where 〈〉 denotes smoothing in both time and frequency, *W*_*x*_(*f*,*τ*) represents the wavelet transform of series *x*(*t*), *W*_*y*_(*f*,*τ*) is the wavelet power transform of series *y*(*t*), and *W*_*x*,*y*_(*f*,*τ*)=*W*_*x*_(*f*,*τ*) · *W*_*y*_(*f*,*τ*) is the cross wavelet power spectrum. The wavelet coherence provides local information about the extent to which two nonstationary signals *x*(*t*) and *y*(*t*) are linearly correlated at a certain period or frequency. *R*_*x*,*y*_(*f*,*τ*) is equal to 1 when there exists a perfect linear relationship at a particular time and frequency between the two signals [22]. The wavelet coherence MATLAB code in [24] was adopted for this study. In the code, the climate variables and mosquito abundance are denoted by *x*(*t*) and the number of malaria cases is denoted by *y*(*t*). The code is developed to handle the seasonality and temporal autocorrelation of the data.

## 3. Results and Discussion

### 3.1. Time Series and Annual Variation of Climate Variables and Malaria Cases

**Figure 2:**
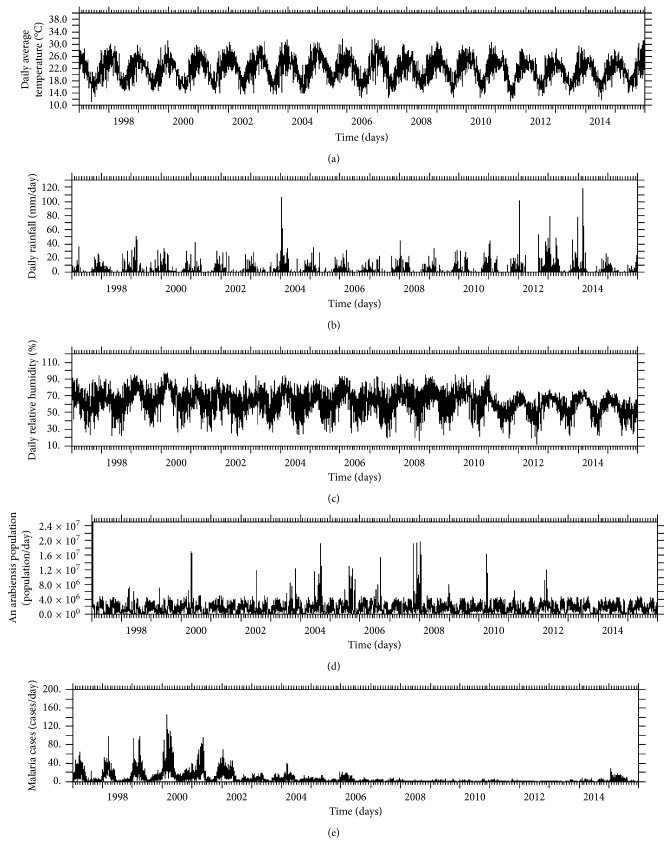
Time series of daily climate variables and malaria cases over the calibration period showing the (a) daily average temperature (°C), (b) rainfall (mm/day), (c) relative humidity (%), (d) simulated *An. arabiensis* population (population/day), and (e) malaria cases of Nkomazi Municipality (cases/day), Mpumalanga Province, South Africa, from January 1997 to December 2015.

In general (according to our time series of daily climate variables), the climatic parameters (temperature, rainfall, and relative humidity) and mosquito abundance vary with malaria cases of Nkomazi Municipality, not surprisingly. All the variables have been shown to peak during the early months of the year from 2004 to 2011 (Figure 2) and are minimal in the middle of the year. However, the peaks are not uniform through the years except for temperature (Figure 2(a)). The peaks were higher (over 100 mm/day) in early 2004 and 2011–2014 for rainfall (Figure 2(b)) and over 90% for relative humidity from early 1997 to 2010 falling below 70% thereafter (Figure 2(c)). Mosquito population (Figure 2(d)) was at its peak in 2000 which corresponds to the malaria outbreak in that same year. Simulated peaks for mosquito abundance in 2007, 2010, and 2012 were inconsistent with malaria cases (Figure 2(d)) as there were almost zero cases observed during these periods. Malaria cases dropped from 2008 through 2014 with a small rise in early 2015. The decrease in cases and the almost zero cases observed in 2007, 2010, and 2012 could be attributed to various malaria control programs which were introduced to the region [2, 5]. The increase in 2015, however, could be as a result of more conducive climatic conditions for vector survival around these periods, coupled with relaxed malaria control strategies [1]. These control strategies were not captured in the mosquito model.

**Figure 3:**
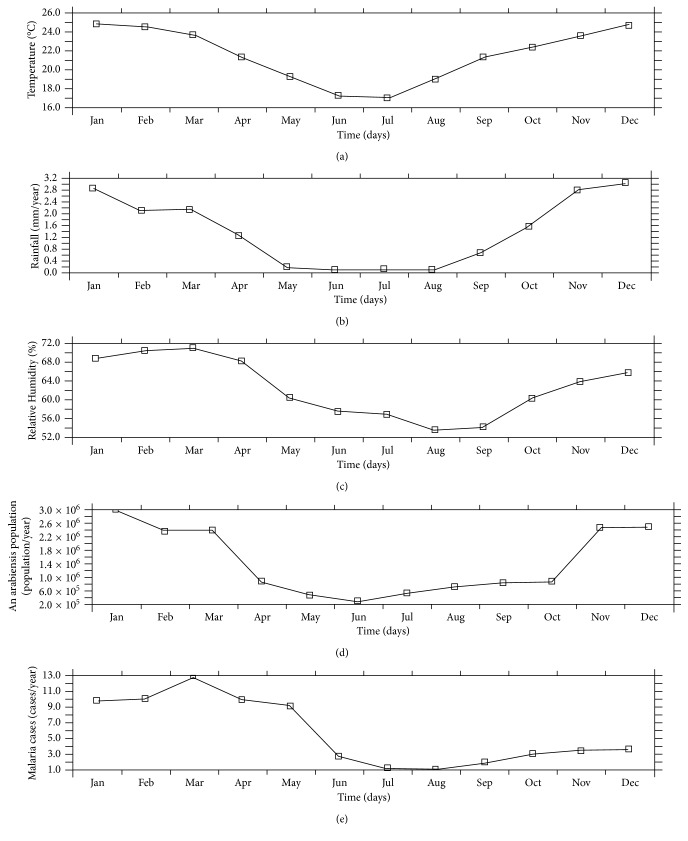
Annual variation of (a) temperature (°C), (b) rainfall (mm), (c) relative humidity (%), (d) *An. arabiensis* population, and (e) malaria cases of Nkomazi Municipality, Mpumalanga Province, South Africa, from January 1997 to December 2015.

The annual variation of temperature, rainfall, relative humidity, mosquito population, and malaria cases offers more detailed information for each month of the year associated with the peaks (Figure 3). The climatic parameters and *An. arabiensis* population show similar curves with malaria cases through the months, but relative humidity shows stronger similarity than the other variables. For instance, relative humidity (Figure 3(c)) and malaria population (Figure 3(e)) peak around 73% and 13 cases, respectively, in March, and at minimal in August every year. Rainfall also shows similar variation (Figure 3(b)). Mosquito abundance (Figure 3(d)) peaks in January. Rainfall is minimum from May to August, while the *Anopheles* population is minimal in June and peaks up thereafter. The variation in temperature is very different as it peaks in December and is minimal in July (Figure 3(a)). The findings here suggest that temperature may be less responsible for malaria transmission in Nkomazi as previously thought.

### 3.2. Cross-Correlation of Climate Variables and Malaria Cases

**Figure 4:**
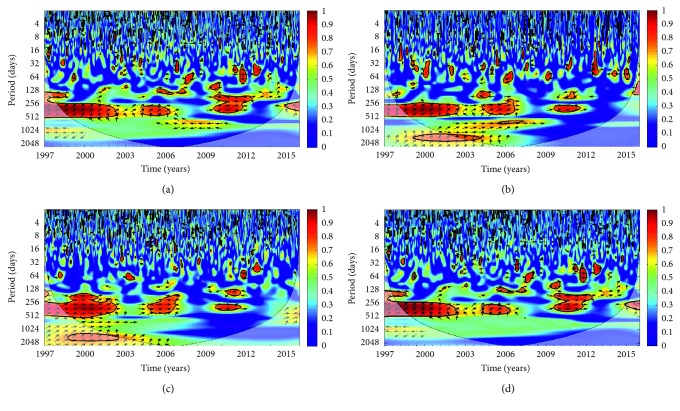
Wavelet coherence analysis showing the correlation and phases between the (a) daily average temperature, (b) rainfall, (c) relative humidity, and (d) *An. arabiensis* population and malaria cases of Nkomazi Municipality, Mpumalanga Province, South Africa, from January 1997 to December 2015. The arrows indicate the relative phasing of the variables, while the faded regions represent the cone of influence and are not considered for the analysis.

Figures 4(a)–4(d) show a significant cross-coherence between the climate variables, mosquito abundance, and malaria occurrence over the municipality. These figures indicate the variability of their relationship over time. In general, malaria shows strong significant coherence with the climatic factors and mosquito abundance especially in the 256–512 days band from the early 1998–2002, 2004–2006, and 2010–2013. However, the coherence is more pronounced on rainfall (Figure 4(b)), relative humidity (Figure 4(c)), and mosquito abundance (Figure 4(d)) than on temperature (Figure 4(a)). For instance, more consistent in-phase (right arrows) relationships are found between malaria and rainfall (Figure 4(b)), relative humidity (Figure 4(c)), and mosquito abundance (Figure 4(d)) than temperature (Figure 4(a)). These phases highlight the positive correlations between the variables and malaria cases and further indicate that the variables always lead to the occurrence of malaria. On the contrary, the noticeable out-phase (left arrows) relationships found between temperature and malaria cases (Figure 4(a)) indicate anticorrelation between the two variables. The findings here are consistent with our findings in Figure 2 even though the figure (Figure 2) failed to give additional information highlighted here.

Several studies have used similar and other analyses to highlight the importance of rainfall on malaria transmission and other infectious diseases. For instance, rainfall has been identified as the major climate variable influencing malaria in western Kenya [28], Tanzania [29], East Africa [30], and Ghana [31]. In addition, the roles of mosquito abundance on malaria transmission have been emphasized in many other studies [9, 32–34]. It should be noted, though, that the significance levels of the different factors influencing malaria occurrence are different for different regions. In some regions in Africa, it was found that temperature may be the primary driver of malaria, for example, in East Africa [35], China [36], Central African Republic, Gabon, Zimbabwe, and Mozambique [18].

### 3.3. Lagged Correlation Analysis between Climate Variables, *An. arabiensis* Population, and Malaria Cases

**Figure 5:**
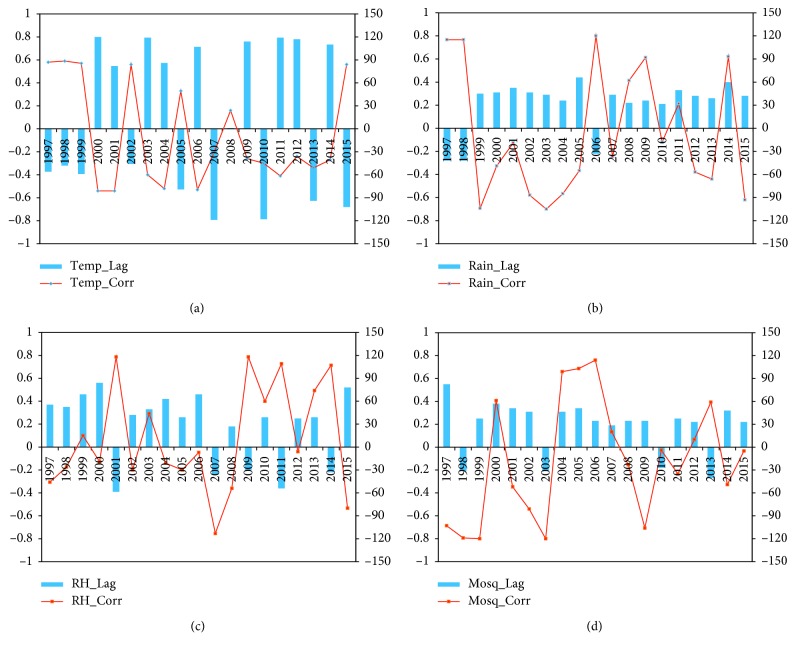
Cross-correlation and time lag of climate variables and malaria cases showing the lag and correlation coefficients between (a) daily temperature, (b) rainfall, (c) relative humidity, and (d) simulated population of *An. arabiensis* and malaria cases of Nkomazi Municipality, Mpumalanga Province, South Africa, from January 1997 to December 2015. Temp_Lag, Rain_Lag, RH_Lag, and Mosq_Lag, respectively, indicate the time lag between the daily temperature, rainfall, relative humidity, and simulated mosquito population and malaria cases, while Temp_Corr, Rain_Corr, RH_Corr, and Mosq_Corr, respectively, represent the correlation between the daily temperature, rainfall, relative humidity, and simulated mosquito population and malaria cases.

The lagged correlation analysis was carried out on temperature, rainfall, relative humidity, the simulated population of *An. arabiensis*, and malaria cases (Figure 5). The results indicate that malaria transmission in Nkomazi Municipality is influenced by the three climatic factors (rainfall, temperature, and relative humidity) as well as mosquito abundance. However, as shown previously (Figures 4(a)–4(d)), the disease is more strongly associated with rainfall, relative humidity, and mosquito abundance than with temperature. For instance, high correlations between 0.5 and 0.8 or between −0.8 and −0.5 are obtained between malaria cases and the three variables: rainfall, relative humidity, and mosquito population, while for temperature the correlation is much weaker (Figure 5(a)). The correlation is more pronounced between malaria cases and rainfall (Figure 5(b)). This is followed by mosquito abundance (Figure 5(d)) and then relative humidity. This shows that temperature is least associated with the occurrence of malaria over the municipality.

Although the findings in Figures 5(a)–5(d)) are consistent with those in Figures 4(a)–4(d)), the previous figures (Figure 4) failed to account for the time lag between the variables and malaria occurrence. Analyses (Figures 5(a)–5(d) show lags of 0 to 90 days for most of the variables. For example, it is seen that malaria transmission occurred 0 to 90 days after it rained in the region. A similar explanation can be offered for mosquito abundance (except for the years 1998, 1999, and 2013). This might be as a result of low rainfall in 1998 and 2013 (Figure 2) and too much rainfall in 2013–2014 which might have washed away some of the larvae [34, 37–41]. In addition, the findings here are consistent with other studies. A time lag of 1–3 months was found between rainfall and malaria cases in Ethiopia [42]. Similar results were found in Eritrea [43]. In particular, Adeola et al. [5] found that rainfall and soil moisture (NDWI) are the major predictors of malaria cases over the Nkomazi Municipality with a lag time of 1–3 months. Their findings also suggested an increase in malaria cases between November and March every year when thresholds for mosquitoes' breeding are met.

As it was noted before, the significance levels of the different factors influencing the transmission of malaria are different for different regions. The findings above show a picture which is different from the findings of studies in other regions. For instance, in [8, 11], it has been found that over KwaZulu-Natal Province, temperature is the primary factor for malaria transmission. This is an indication that factors influencing the transmission of malaria cannot be generalized over South Africa [5]. Although our findings have shown significant correlations between the factors and malaria cases, rainfall is found to be a more influencing factor than temperature. More importantly, these climatic factors and other factors which are not considered in this study are responsible for malaria transmission in South Africa. Running a mosquito model with future climate data over each epidemic region could help predict likely years of malaria outbreak through simulated mosquito abundance.

## 4. Conclusions

In this study, the impact of daily climate variables and abundance of *An. arabiensis* on malaria transmission in Nkomazi Local Municipality was investigated. Over the period 1997–2015, the daily climatic parameters (rainfall, temperature, and relative humidity) and malaria cases of five epidemic villages in the municipality were considered. Since it is essential to consider a long-term data series for this study, the mathematical model presented in [17] was used to simulate the population dynamics of *An. arabiensis*. The interconnection, correlation, and lag time between malaria cases and the other variables were further examined.

The results highlight the importance of the climatic parameters and abundance of mosquitoes on malaria transmission over the municipality. In particular, rainfall was confirmed as the major driver of malaria over the region, followed by an abundance of *An. arabiensis* and relative humidity. Temperature was found to be less significant on the transmission of malaria in the municipality over the study period. Results from the wavelet analysis further ascertained the closer relationship between mosquito population and rainfall than temperature. It is also found that an average lag time of 0 to 90 days is required between most of the variables and malaria transmission in the municipality. We would also like to conclude that the significance levels of climatic factors influencing the occurrence of malaria are different for different regions as highlighted in the previous section and other studies [18, 28–31, 35, 36].

The findings from this study would be suitable for an early-warning system or prediction of malaria transmission over the study region or entire province. With a proper understanding of how climate affects malaria risk and noting the lag of malaria cases with respect to climatic conditions, it is possible to identify future high-risk situations.

The present study has considered a dynamic mosquito model for the simulations of mosquito abundance. The climate-based model incorporates temperature and rainfall as the input parameters. Other factors such as migratory movements, prophylactic and hygienic-sanitary treatments, and land use have been identified as significant factors in the transmission of malaria and mosquito abundance. Quality of drainage which determines the stability, availability, and productivity of the vector breeding habitats has also been highlighted as another key factor influencing malaria transmission [44]. Poor drainage can produce stable breeding habitats for mosquitoes; hence, less rainfall may be required over the study region. Incorporating some of these factors into the model could offer more precise outputs. However, the current study leaves these aspects for future studies.

## Data Availability

The malaria data reported in this manuscript have been sourced from the provincial Integrated Malaria Information System (IMIS) of Malaria Control Programme in the Mpumalanga Provincial Department of Health and were obtained from the South African Weather Service (SAWS) through its collaborative research with the University of Pretoria Institute for Sustainable Malaria Control (UP ISMC). The climate data were obtained from the National Center for Environmental Prediction (NCEP) Climate Forecast System Reanalysis (CFSR) and the Tropical Rainfall Measuring Mission (TRMM).
